# The diagnostic and prognostic value of soluble ST2 in Sepsis

**DOI:** 10.3389/fmed.2024.1487443

**Published:** 2024-11-21

**Authors:** Xinghua Ye, Jia Wang, Le Hu, Ying Zhang, Yixuan Li, Jingchao Xuan, Silu Han, Yifan Qu, Long Yang, Jun Yang, Junyu Wang, Bing Wei

**Affiliations:** Emergency Medicine Clinical Research Center, Beijing Key Laboratory of Cardiopulmonary Cerebral Resuscitation, Beijing Chao-Yang Hospital, Capital Medical University, Beijing, China

**Keywords:** APACHE II score, diagnosis, lactic acid, prognosis, Sepsis, SOFA score, soluble ST2

## Abstract

**Objective:**

To determine the diagnostic and prognostic value of soluble suppression of tumorigenicity 2 (sST2) in patients with sepsis.

**Methods:**

A total of 113 critically ill patients were enrolled at the emergency department of Beijing Chaoyang Hospital Jing Xi Branch. Venous blood levels of sST2 were measured using the AFIAS-6 dry fluorescence immunoassay analyzer. Based on Sepsis 3.0 criteria, patients were categorized into a sepsis group (76 cases) and a non-sepsis group (37 cases). The sepsis group was further divided into non-survivors (38 cases) and survivors (38 cases) based on 28-day survival outcomes. The vital signs, blood gas analysis, routine blood tests, liver and kidney function tests, procalcitonin (PCT), C-reactive protein (CRP), sST2, left ventricular ejection fraction (LVEF), and other basic characteristics of the patients were recorded. Further, the SOFA, qSOFA and APACHE II scores of each patient were calculated. Statistical analysis was performed using SPSS 25.0, including logistic regression and ROC curve analysis to assess prognostic factors.

**Results:**

The serum sST2 levels in the sepsis group (125.00 ± 60.32 ng/mL) were significantly higher than in the non-sepsis group (58.55 ± 39.03 ng/mL) (*p* < 0.05). The SOFA score (8.08 ± 2.88), APACHE II score (18.00 ± 4.72), blood sST2 levels (168.06 ± 36.75 ng/mL) and lactic acid levels (2.89 ± 3.28) in the non-survivor group were significantly higher than the survivor group (*p* < 0.05). Multiple logistic regression analysis showed that sST2, SOFA score, APACHE II score and lactic acid levels were independent risk factors for poor prognosis in patients with sepsis. The ROC curve analysis of the above indexes showed no significant differences between the AUC of sST2 (0.912) and the SOFA score (0.929) (*z* = 0.389, *p* = 0.697), or the APACHE II score (0.933) (*z* = 0.484, *p* = 0.627). However, there was a significant difference between the AUC of sST2 (0.912) and lactic acid levels (0.768) (*z* = 2.153, *p* = 0.030).

**Conclusion:**

Blood levels of sST2 show a clinically diagnostic and prognostic value in sepsis. Further, sST2 shows a similar predictive ability as the SOFA and APACHE II scores in determining the prognosis of sepsis patients. However, sST2 has a higher predictive ability than lactic acid levels in determining prognosis in sepsis.

## Introduction

Sepsis, a life-threatening condition characterized by a dysregulated host response to infection, remains a significant contributor to mortality rates in intensive care units worldwide, affecting approximately 20–30% of individuals hospitalized in these critical care settings (1). Despite advances in critical care, the early diagnosis and accurate prognosis of sepsis continue to pose significant challenges (2). Accurate biomarkers that can facilitate early detection and provide reliable prognostic information are urgently needed to improve patient outcomes.

Interleukin 1 receptor-like 1, also known as Suppression of tumorigenicity 2 (ST2), is a member of the interleukin-1 (IL-1) receptor family. In recent years, ST2 has attracted attention as a new marker in heart failure and inflammation (3). ST2, a specific receptor for IL-33 within the IL-1 receptor family, plays a crucial role in immune regulation and systemic inflammatory responses (4–6). It exists in four isoforms: transmembrane ST2L, soluble sST2, truncated ST2v, and long variant ST2LV. sST2 (soluble ST2) competitively binds to IL-33, preventing its interaction with membrane-bound ST2 and inhibiting subsequent signaling. In cases of severe infections, sST2 functions as a negative regulator by binding to IL-33, thereby contributing to immunosuppression (7, 8). Several studies have reported that the IL-33 / ST2 signaling pathway is crucial in various inflammatory diseases, cancer, and heart diseases (9–11). However, only a few studies have investigated the role of ST2 in sepsis. Given the pivotal role of inflammation in the pathophysiology of sepsis, the potential utility of soluble ST2 (sST2) as a diagnostic and prognostic biomarker warrants thorough investigation. This study analyzed the blood levels of sST2 in acute and critically ill patients. Further, the study also explored the diagnostic and prognostic role of sST2 in sepsis patients. By shedding light on the diagnostic and prognostic value of sST2 in sepsis, this study contributes to the broader effort to improve outcomes in this challenging and often fatal condition.

## Materials and methods

### Study population

**Figure 1:**
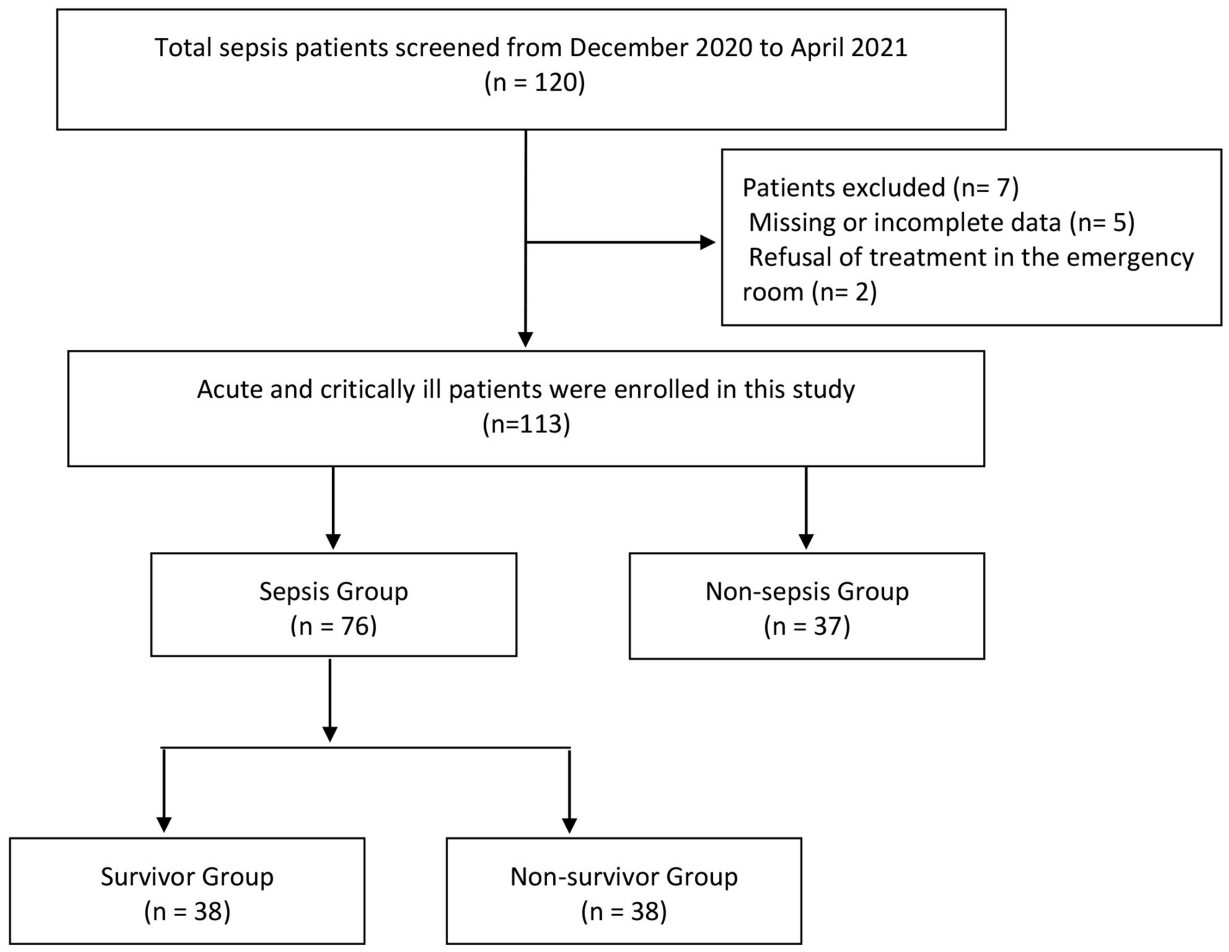
The flowchart of the patient screening process.

A total of 120 sepsis patients were screened from December 2020 to April 2021. Of these, 7 patients were excluded: 5 due to missing or incomplete data and 2 who refused treatment in the emergency room. Consequently, 113 acute and critically ill patients were prospectively enrolled in the emergency department at the Beijing Chaoyang Hospital Jing Xi Branch during this period. Inclusion criteria were: (a) patients aged ≥18 years and (b) patients with a diagnosis of infectious diseases during the admission period from December 2020 to April 2021. Exclusion criteria included: (a) age < 18 years, (b) missing or incomplete patient data, and (c) refusal to be managed in the emergency department. The flowchart of the patient screening process is presented in Figure 1. The patients included 53 males and 60 females, aged between 33 and 94 years. Sepsis and septic shock (hereinafter referred to as “sepsis 3.0”) were diagnosed based on the international consensus on the definition of sepsis published by the European Society of Critical Care Medicine in 2016 (12). The patients were then classified into the sepsis group (*n* = 76) and the non-sepsis group (*n* = 37). Patients in the sepsis group were further subdivided based on the outcome after 28 days into the non-survivor group (38 cases) and the survivor group (38 cases). Routine diagnostic tests were conducted, and treatment was optimized based on the outcomes of the tests. Data on the vital signs, routine blood tests, liver and kidney function tests, blood gas analysis, C-reactive protein (CRP), procalcitonin (PCT), and cardiopulmonary function were recorded. Collected vital signs included body temperature, heart rate, respiratory rate, and mean arterial pressure (MAP). Routine blood tests comprised white blood cell count (WBC), hemoglobin level (HB), hematocrit (HCT), platelet count (PLT), and liver function tests such as aspartate aminotransferase (AST), alanine aminotransferase (ALT), total bilirubin (TBIL), and albumin (ALB). Kidney function tests included blood urea nitrogen (BUN) and creatinine (CR) measured from blood serum samples. Blood gas analysis included pH, partial pressure of oxygen (PaO2), partial pressure of carbon dioxide (PaCO2), and lactate level, all determined from arterial blood samples. Cardiopulmonary function parameters included ejection fraction (EF) (%) and oxygenation index, which were measured using echocardiography and blood gas analysis, respectively. These parameters were measured and assessed immediately upon the patient’s arrival in the emergency room. The SOFA and APACHE II scores were subsequently calculated based on the collected data. sST2 was measured within 72 h of admission. Further, patient’s survival was followed up for 28 days. This study obtained the informed consent of all patients and their families, signed the informed consent form, and was approved by the ethics committee of Beijing Chao Yang Hospital, Capital Medical University (number: 2020-6-17-2).

### ST2 detection by immunofluorescence

Venous blood was collected within 2 h of the patient appearing in the emergency department. The samples were collected into purple capped tubes lined with K2-EDTA anticoagulant. ST2 was analyzed using an automated immunofluorescence immunoassay system (AFIAS) immune analyzer (Model: AFIAS-6, Origin: Korea) and AFIAS ST2 Kit (REF: SMFP-70, Origin: Korea). All methods were performed in accordance with the relevant guidelines and regulations in the methods section to this effect.

### Statistical analysis

Statistical analysis was conducted using the statistical software SPSS 25.0. The normal distribution of data was assessed using the nonparametric Kolmogorov–Smirnov test. Data were expressed as mea*n* ± standard deviation ( $\bar{x\;}$ ±s) for normally distributed data or median and interquartile range for not normally distributed data. Differences in qualitative parameters between groups were assessed using two independent sample *t*-test (for normally distributed data). In contrast, the Mann Whitney U test was used for comparisons between groups (for not normally distributed data). On the other hand, one-way ANOVA was used for comparison between multiple groups. Categorical variables were expressed as numbers, and the data were analyzed using the chi-square test. Correlation between variables was conducted using Spearman correlation coefficients. The logistic regression model was used to analyze the prognostic factors. The receiver operating characteristic curve (ROC curve) was plotted to evaluate factors affecting patient prognosis. Statistically significant differences were considered at a *p*-value < 0.05.

## Results

### Comparison of the general information

There were 76 patients in the sepsis group, with an average age of 80.75 years, including 33 males and 43 females. However, there were 37 patients in the non-sepsis group, with an average age of 74.38 years, including 20 males and 17 females. There was no statistically significant difference in gender and age between the sepsis group and the non-sepsis group (all *p* > 0.05) (Table 1). Vital signs showed no significant difference in heart rate, mean arterial pressure, and body temperature between the sepsis and non-sepsis groups (all *p* > 0.05). The respiratory rate was significantly higher in the sepsis group (*p* = 0.021). Laboratory findings revealed no significant differences in white blood cell counts and platelet counts between the two groups (all *p* > 0.05). In contrast, hemoglobin levels were significantly reduced in the sepsis group (*p* = 0.014). Additionally, liver enzyme levels (AST and ALT) were comparable across both groups, with all *p*-values greater than 0.05. The baseline characteristics of the sepsis group and the non-sepsis group are summarized in Table 1.

**Table 1:** Comparison of baseline characteristics between the sepsis group and non-sepsis group.

Detection indexes	Sepsis group (*n* = 76)	Non-sepsis group (*n* = 37)	*p-*value
Age (years)	80.75 ± 8.90	74.38 ± 8.95	0.782
Male/Female	33/43	20/17	0.65
SOFA score	5.71 ± 3.35	2.22 ± 2.32	0.005
APACHE II score	14.34 ± 5.46	9.68 ± 4.53	0.282
Temperature (°C)	36.53 ± 0.44	36.47 ± 0.37	0.441
Heart rate (Bpm)	90.34 ± 21.07	88.43 ± 20.50	0.649
Respiratory rate (breaths/min)	26.70 ± 8.90	22.65 ± 8.40	0.021
Mean arterial pressure (mmHg)	92.16 ± 14.56	92.06 ± 14.37	0.973
pH	7.43 ± 0.09	7.44 ± 0.07	0.35
PaCo2 (mmHg)	42.97 ± 11.88	40.05 ± 7.18	0.108
PaO2 (mmHg)	92.28 ± 47.19	95.00 ± 37.95	0.742
Lactic acid (mmol/l)	2.03 ± 2.49	1.43 ± 1.14	0.079
White blood cells (10^9^/L)	9.51 ± 4.75	9.34 ± 4.26	0.845
Platelets (10^9^/L)	208.78 ± 69.01	213.01 ± 98.04	0.792
Hematocrit (%)	40.52 ± 27.87	32.27 ± 6.80	0.084
Hemoglobin (g/L)	104.83 ± 21.83	120.14 ± 33.28	0.014
Aspartate aminotransferase (U/L)	37.54 ± 49.30	40.75 ± 43.13	0.724
Alanine aminotransferase (U/L)	30.12 ± 37.39	28.53 ± 32.80	0.818
Total bilirubin (μmol/L)	16.56 ± 11.02	16.46 ± 8.21	0.958
Albumin (g/L)	27.38 ± 6.15	28.53 ± 32.80	0.014
Blood urea nitrogen (mg/dL)	16.27 ± 13.69	10.78 ± 11.12	0.025
Creatinine (μmol/L)	115.88 ± 110.40	100.16 ± 170.76	0.612
Sodium (mEq/L)	140.61 ± 9.00	138.07 ± 6.50	0.09
Potassium (mEq/L)	4.03 ± 0.71	4.02 ± 0.62	0.899
PCT (ng/ml)	2.01 ± 4.59	0.90 ± 2.75	0.095
CRP (mg/l)	49.22 ± 44.76	37.22 ± 44.55	0.555
BNP (pg/ml)	584.52 ± 772.13	474.27 ± 741.99	0.52
TnI (ng/ml)	0.15 ± 0.64	1.36 ± 4.87	≤0.001
LVEF (%)	60.50 ± 11.33	60.24 ± 12.54	0.239
Hospital stay (days)	14.64 ± 9.58	11.97 ± 8.30	0.135
sST2 (ng/ml)	125.00 ± 60.32	58.55 ± 39.03	≤0.001

Values are expressed as the mean ± standard deviation. APACHE II, acute physiology and chronic health evaluation II; BNP, B-type natriuretic peptide; CRP, C-reactive protein; LVEF, left ventricular ejection fraction; PCT, procalcitonin; SOFA, sequential organ failure assessment; sST2, soluble suppression of tumorigenicity 2; TnI, troponin I.

### Comparison of sST2 values between the sepsis group and the non-sepsis group

The sST2 in venous blood of patients in the sepsis group was higher than in the non-sepsis group, with a statistically significant difference (Table 1). The Spearman correlation analysis showed that sST2 was positively correlated with the SOFA score (*r* = 0.539, *p* ≤ 0.001) and APACHE II score (*r* = 0.482, *p* ≤ 0.001).

### Prognosis prediction in sepsis patients using sST2 and other laboratory parameters

There were no statistically significant differences in age, gender, brain natriuretic peptide (BNP), troponin I (TnI), LVEF, and length of hospital stay between the non-survivor group and the survivor group (all *p* > 0.05) (Table 2). However, the non-survivor group’s SOFA score, APACHE II score, lactic acid, PCT, CRP, and sST2 levels were significantly higher than the survivor group (all *p* < 0.05).

**Table 2:** Comparison of the detection indexes between the non-survivor group and the survivor group of sepsis patients.

Detection indexes	Non-survivor group (*n* = 38)	Survivor group (*n* = 38)	*p-value*
Male/Female	13/25	20/18	0.108
Age (years)	81.61 ± 7.93	79.89 ± 9.81	0.406
SOFA score	8.08 ± 2.88	3.34 ± 1.71	≤0.001
APACHE II score	18.00 ± 4.72	10.68 ± 3.26	≤0.001
Lactic acid (mmol/l)	2.89 ± 3.28	1.16 ± 0.52	0.010
PCT (ng/ml)	3.65 ± 6.07	0.36 ± 0.64	0.001
CRP (mg/l)	66.85 ± 45.38	31.59 ± 36.93	≤0.001
BNP (pg/ml)	666.78 ± 697.17	502.26 ± 841.74	0.356
TnI (ng/ml)	0.23 ± 0.90	0.08 ± 0.08	0.305
LVEF (%)	60.13 ± 8.74	60.87 ± 13.55	0.779
Hospital stay (days)	16.45 ± 10.85	12.84 ± 7.87	0.101
sST2 (ng/ml)	168.06 ± 36.75	81.93 ± 47.08	≤0.001

Values are expressed as the mea*n* ± standard deviation. APACHE II, acute physiology and chronic health evaluation II; BNP, B-type natriuretic peptide; CRP, C-reactive protein; LVEF, left ventricular ejection fraction; PCT, procalcitonin; SOFA, sequential organ failure assessment; sST2, soluble suppression of tumorigenicity 2; TnI, Troponin I.

**Figure 2:**
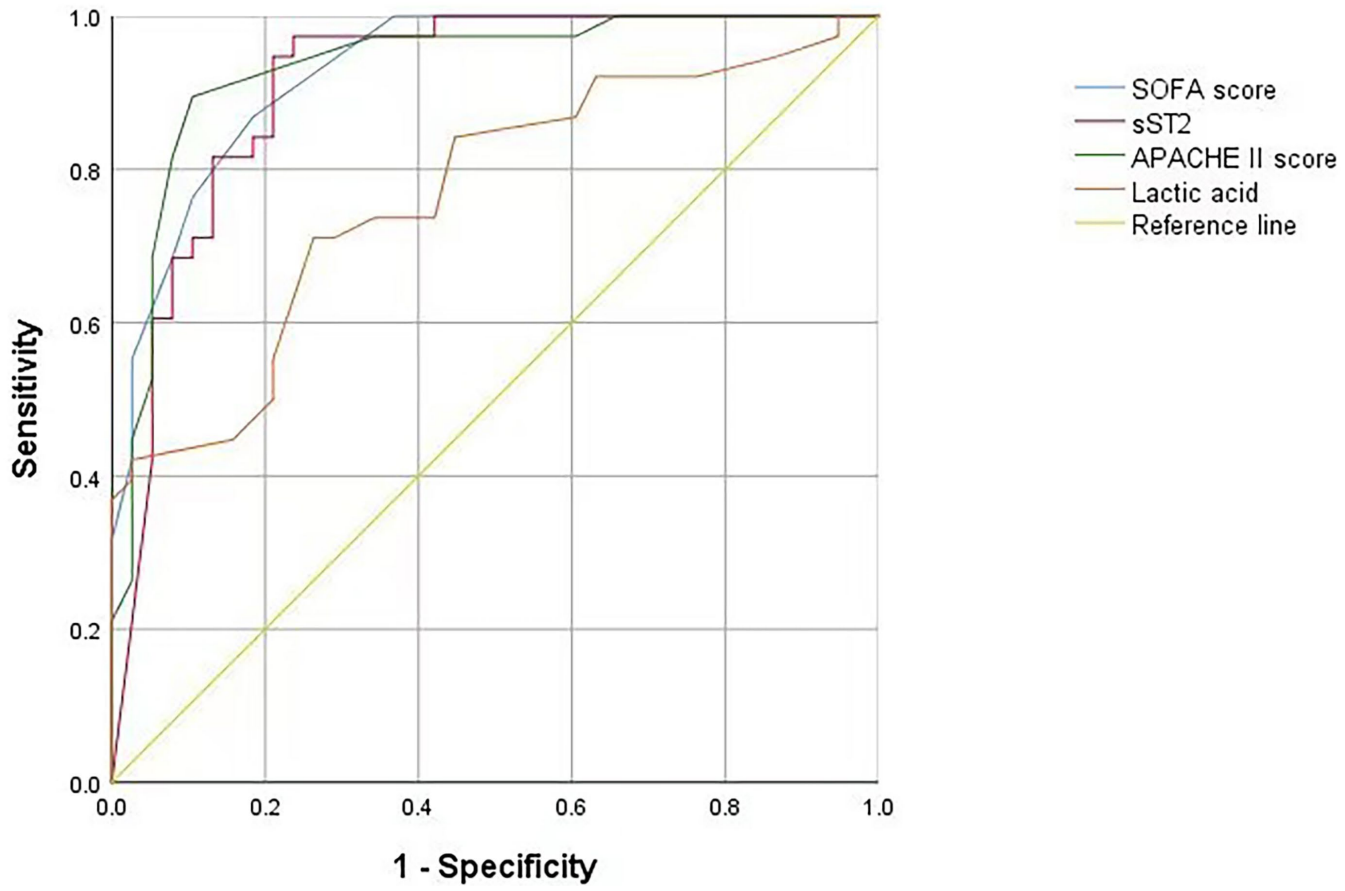
ROC curve of the SOFA score, sST2, APACHE II score and lactic acid levels on the prediction of mortality. ROC curve, receiver operating characteristic curve; SOFA score, sequential organ failure assessment score. sST2, soluble suppression of tumorigenicity 2; APACHE II, acute physiology and chronic health evaluation II.

**Figure 3:**
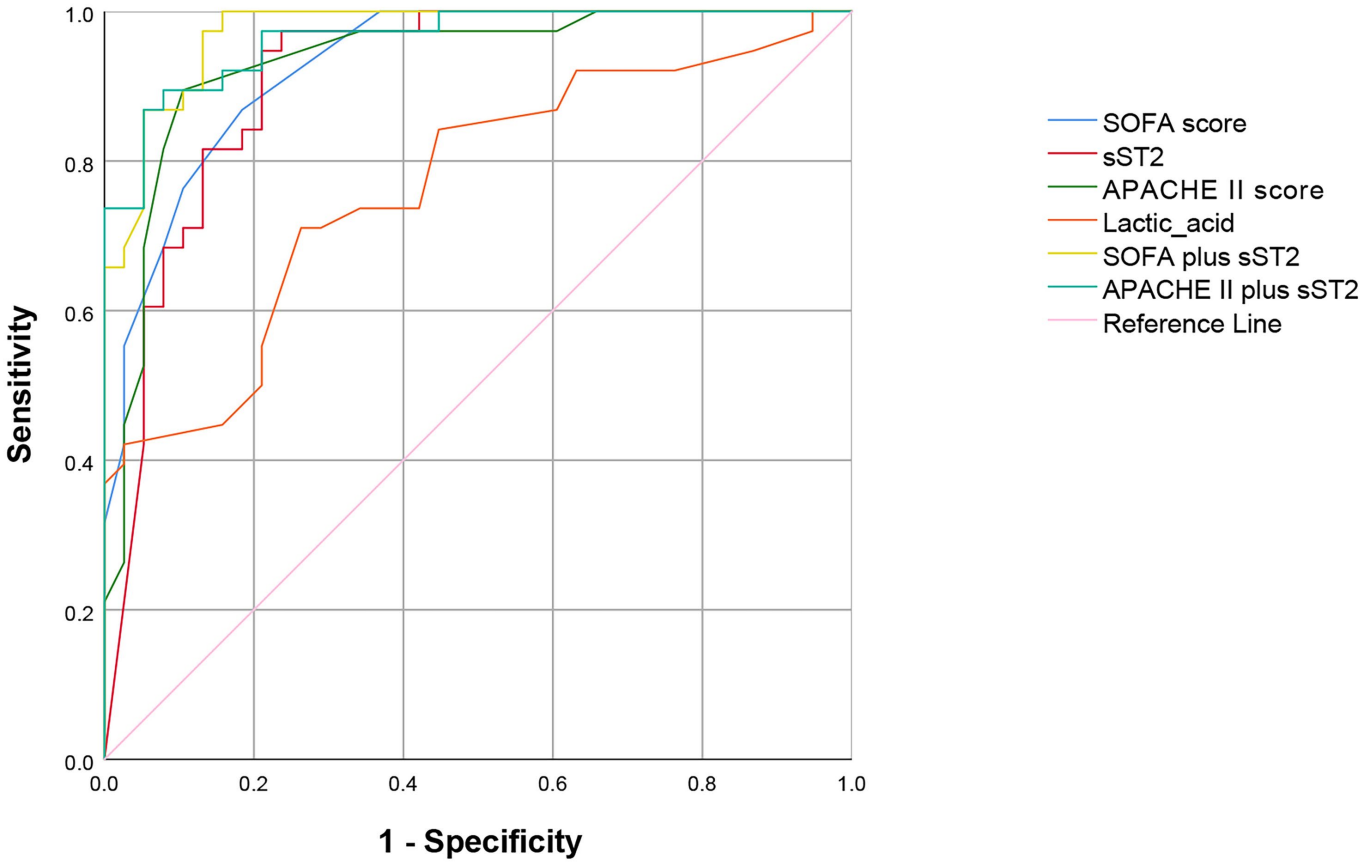
ROC curve showing the predictive value of combining SOFA with sST2 and APACHE II with sST2 for mortality prediction. ROC curve, receiver operating characteristic curve; SOFA score, sequential organ failure assessment score. sST2, soluble suppression of tumorigenicity 2; APACHE II, acute physiology and chronic health evaluation II.

Multivariate logistic regression analysis of the statistically significant prognostic factors in the univariate analysis showed that sST2, SOFA score, APACHE II score, and lactic acid levels were independent prognostic factors for sepsis (Table 3). Analysis of the ROC curve showed that the area under the curve (AUC) of the sST2 and SOFA score (0.912 vs. 0.929) (*z* = 0.389, *p* = 0.697), and the area under the curve of the sST2 and Apache II score were not statistically significant (0.912 vs. 0.933) (*z* = 0.484, *p* = 0.627) (Figure 2 and Table 4). However, the AUC of sST2 and lactic acid levels was statistically significant (0.912 vs. 0.768) (*z* = 2.153, *p* = 0.030). sST2 showed a sensitivity, specificity, positive predictive value (PPV), negative predictive value (NPV), positive likelihood ratio (+LR), and a negative likelihood ratio (−LR) of 97.4%, 76.3%, 80.4%, 96.7%, 4.11, and 0.03, respectively, in predicting the prognosis of sepsis. The SOFA score had a sensitivity, specificity, PPV, NPV, +LR, and –LR of 86.8%, 81.6%, 82.5%, 86.1%, 4.71, and 0.16, respectively, in predicting the prognosis of sepsis. The APACHE II score had a sensitivity, specificity, PPV, NPV, +LR, and –LR of 89.5%, 89.5%, 89.5%, 89.5%, 8.5, and 0.12, respectively, in predicting the prognosis of sepsis. The lactic acid levels had a sensitivity, specificity, PPV, NPV, +LR, and –LR of 71.1%, 73.7%, 73.0%, 71.8%, 2.7 and 0.39, respectively, in predicting the prognosis of sepsis. In summary, sST2 demonstrated prognostic and predictive ability comparable to the SOFA and APACHE II scores in sepsis, and it showed higher predictive ability than lactic acid levels. Moreover, ROC curve analysis revealed that the combination of SOFA with sST2 achieved the highest AUC of 0.973, indicating superior distinguishing ability for predicting outcomes in sepsis (Figure 3). This was closely followed by the combination of APACHE-II with sST2, with an AUC of 0.964. However, the results indicated that the combinations of SOFA with sST2 and APACHE-II with sST2 had similar distinguishing abilities, as there was no statistically significant difference between their AUCs (*z* = 0.496, *p* = 0.620) (Figure 3).

**Table 3:** Multivariate logistic regression analysis of the factors affecting prognosis in sepsis patients.

Detection indexes	Standard error	Wald	Sig.	EXP (B)	95% CI of EXP(B)	
					Lower limit	Upper limit
SOFA score	0.529	6.334	0.046	0.349	0.124	0.984
APACHE II score	0.237	3.888	0.049	0.626	0.394	0.997
Lactic acid	1.850	3.954	0.047	0.025	0.001	0.949
PCT	1.094	0.003	0.959	0.945	0.111	8.061
CRP	0.017	0.085	0.771	1.005	0.973	1.038
sST2	0.029	4.337	0.037	0.941	0.889	0.996

APACHE II, acute physiology and chronic health evaluation II; CRP, C-reactive protein; Lactic acid, lactic acid; PCT, procalcitonin; sST2, soluble suppression of tumorigenicity 2; SOFA, sequential organ failure assessment.

**Table 4:** Diagnostic parameters of the SOFA score, sST2, APACHE II score and lactic acid levels.

Detection indexes	AUC	Cut-off	Sensitivity (%)	Specificity (%)	PPV (%)	NPV (%)	+LR	-LR
sST2	0.912	103.055	97.4	76.3	80.4	96.7	4.11	0.03
SOFA score	0.929	4.5	86.8	81.6	82.5	86.1	4.71	0.16
APACHE II score	0.933	13.5	89.5	89.5	89.5	89.5	8.5	0.12
Lactic acid	0.768	1.5	71.1	73.7	73.0	71.8	2.7	0.39

APACHE II, acute physiology and chronic health evaluation II; Lactic acid, lactic acid; SOFA, sequential organ failure assessment; sST2, soluble suppression of tumorigenicity 2.

## Discussion

Sepsis is a life-threatening organ dysfunction caused by an imbalance in the body’s response to infection, leading to septic shock or multiple organ dysfunction (13). Sepsis is a medical emergency that presents as an acute and severe disease. It is associated with high mortality, which can be as high as 40% (14). The occurrence and development of sepsis involve complex immune mechanisms (15, 16). Sepsis is characterized by an inflammatory storm in the early stages and persistent immunosuppression in later stages. Further, it is characterized by reduced innate and acquired immune response and reduced ability for pathogen clearance, resulting in secondary opportunistic infections by pathogenic bacteria or viruses and severe complications (17).

ST2 is a specific receptor of IL-33 in the IL-1 family. IL-33 / ST2 signaling pathway plays an important role in the systemic inflammatory response and immune regulation (4–6, 18). ST2 includes four isoforms, ST2L, sST2, ST2v, and ST2LV. sST2 is a soluble ST2 that can competitively bind to IL-33, inhibiting its biological activity and signal transduction. In severe infection, sST2 acts as a negative regulator and combines with IL-33, thus participating in immunosuppression (7, 8). In the present study, the sST2 levels in the venous blood were higher in the sepsis group than in the non-sepsis group. This finding indicates that sST2 can be used as a diagnostic index in sepsis.

Moreover, in the sepsis group, the blood levels of sST2 were significantly higher in the non-survivor group than in the survivor group, suggesting that the blood levels of sST2 have a high predictivity ability in determining the prognosis of sepsis patients. Higher blood levels of sST2 were positively correlated with a poor prognosis. Furthermore, patients with high SOFA and APACHE II scores also had high blood levels of sST2, with a poorer prognosis, consistent with other studies (19, 20). Therefore, blood levels of sST2 in patients with sepsis can be used as clinical indicators to predict prognosis.

APACHE II scoring system has been widely used in ICUs since its inception in 1985 (21–24). It is of clinical significance as it can objectively evaluate the severity of the patient’s condition, guide the monitoring and treatment plans, and evaluate treatment outcomes (25). Furthermore, it can be used to predict and accurately assess the quality of care in ICU settings.

The SOFA score was described by the European Society of Intensive Care Medicine in 1944. The score aims to describe the occurrence and development of multiple organ dysfunction syndromes (MODS) and to evaluate the incidence rate (23, 24). The SOFA score is based on objective, simple, easy-to-obtain, reliable, and specific continuous variables in evaluating multiple organ dysfunction (26). Patient source, disease type, demographic characteristics, and the treatment administered do not influence these variables. The SOFA score can distinguish the degree of multiple organ dysfunction or failure of a single organ (26).

Lactic acid is a metabolite of anaerobic glycolysis in the human body. Under normal circumstances, levels of lactic acid exceeding 2 mmol/L overwhelm the capacity for liver clearance (27, 28). The dynamic monitoring of blood lactate levels is clinically significant in diagnosing lactic acidosis (29). Increased blood lactate levels can be used to evaluate disease severity and prognosis (27, 28).

The ROC curve analysis showed that sST2, SOFA, and APACHE II scores and the lactic acid levels had a prognostic, predictive ability in sepsis, consistent with previous studies. The sST2 showed similar prognostic and predictive ability with the SOFA and APACHE II scores. However, sST2 had a higher prognostic predictive ability than lactic acid levels. In conclusion, blood levels of sST2 can be used as clinical indices for the diagnosis and prognosis of sepsis.

Our study has several limitations. First, while sST2 demonstrates significant diagnostic and prognostic utility in sepsis, its performance must be validated through more extensive prospective cohort studies with a more diverse patient population. Second, the single-center design may limit the generalizability of our results to other clinical settings. Therefore, multi-center studies are warranted to corroborate the findings across various clinical environments. Third, although the 28-day follow-up period helps assess short-term outcomes, it may not capture the long-term prognostic significance of sST2. Further research is needed to explore this aspect. Fourth, we treated sepsis and septic shock as a homogeneous entity. While our primary focus was to assess the prognostic value of soluble ST2 across the full spectrum of sepsis, this approach may mask differences in outcomes associated with these distinct clinical phenotypes. Future studies should consider applying the Sepsis-3 criteria to provide deeper insights into the differential roles of soluble ST2 in sepsis and septic shock. Finally, while the study compared sST2 with SOFA, APACHE II, and lactic acid levels, additional comparisons with other biomarkers, such as procalcitonin and C-reactive protein, could provide further insights.

## Conclusion

In conclusion, this study demonstrates that sST2 has significant diagnostic and prognostic value in sepsis. The predictive ability of sST2 is comparable to established scoring systems like SOFA and APACHE II, which are widely used for determining sepsis prognosis. Notably, sST2 demonstrates superior predictive capability compared to lactic acid levels for sepsis outcomes, suggesting that sST2 could be a more reliable indicator for identifying patients at higher risk of poor prognosis. These findings support the potential incorporation of sST2 into routine clinical practice for more accurate diagnosis and prognosis of sepsis. Further research is needed to validate these results and explore the practical applications of sST2 in sepsis management.

## Data Availability

The raw data supporting the conclusions of this article will be made available by the authors, without undue reservation.
